# The Role of Antimicrobial Peptides as Antimicrobial and Antibiofilm Agents in Tackling the Silent Pandemic of Antimicrobial Resistance

**DOI:** 10.3390/molecules27092995

**Published:** 2022-05-06

**Authors:** Bruno S. Lopes, Alfizah Hanafiah, Ramesh Nachimuthu, Saravanan Muthupandian, Zarith Nameyrra Md Nesran, Sandip Patil

**Affiliations:** 1 Department of Medical Microbiology, School of Medicine, Medical Sciences and Nutrition, University of Aberdeen, Aberdeen AB25 2ZD, UK; 2 Department of Medical Microbiology and Immunology, Faculty of Medicine, Universiti Kebangsaan Malaysia, Kuala Lumpur 56000, Malaysia; zarithnesran@ymail.com; 3 Antibiotic Resistance and Phage Therapy Laboratory, Department of Biomedical Sciences, Vellore Institute of Technology, School of Bioscience and Technology, Vellore 632014, India; drpnramesh@gmail.com; 4 AMR and Nanotherapeutics Laboratory, Department of Pharmacology, Saveetha Institute of Medical and Technical Sciences, Saveetha Dental College, Chennai 600077, India; bioinfosaran@gmail.com; 5 Department of Hematology and Oncology, Shenzhen Children’s Hospital, Shenzhen 518038, China; sandippatil1309@yahoo.com

**Keywords:** antimicrobial peptides, biofilms, Gram-negative bacteria, WHO priority pathogens, ESKAPE, antimicrobial resistance, public health

## Abstract

Just over a million people died globally in 2019 due to antibiotic resistance caused by ESKAPE pathogens (*Enterococcus faecium*, *Staphylococcus aureus*, *Klebsiella pneumoniae*, *Acinetobacter baumannii*, *Pseudomonas aeruginosa*, and *Enterobacter* species). The World Health Organization (WHO) also lists antibiotic-resistant *Campylobacter* and *Helicobacter* as bacteria that pose the greatest threat to human health. As it is becoming increasingly difficult to discover new antibiotics, new alternatives are needed to solve the crisis of antimicrobial resistance (AMR). Bacteria commonly found in complex communities enclosed within self-produced matrices called biofilms are difficult to eradicate and develop increased stress and antimicrobial tolerance. This review summarises the role of antimicrobial peptides (AMPs) in combating the silent pandemic of AMR and their application in clinical medicine, focusing on both the advantages and disadvantages of AMPs as antibiofilm agents. It is known that many AMPs display broad-spectrum antimicrobial activities, but in a variety of organisms AMPs are not stable (short half-life) or have some toxic side effects. Hence, it is also important to develop new AMP analogues for their potential use as drug candidates. The use of one health approach along with developing novel therapies using phages and breakthroughs in novel antimicrobial peptide synthesis can help us in tackling the problem of AMR.

## 1. Introduction

The discovery of penicillin in the year 1928 was one of the most important scientific discoveries in modern medicine. It was possible only because Sir Alexander Fleming noticed a clear zone around an invading fungus on an agar plate, leading to the inhibition of bacteria, after arriving back to his London laboratory from a two-week vacation. Although this fungus has been identified by various names, such as *Penicillium rubrum*, *P. notatum*, and *P. chrysogenum*, recent genomic and phylogenetic analyses show that it is *P. rubens* [1]. Penicillin F was the purified compound isolated in 1940 by a team led by Howard Florey and Ernst Chain at the University of Oxford. For this discovery, Florey and Chain, along with Fleming, shared the 1945 Nobel Prize in Physiology or Medicine. One important observation made by Fleming was that many bacteria were not affected by penicillin [2]. The 1958 Nobel Prize winner Joshua Lederberg said, “The future of humanity and microbes likely will unfold as episodes of Our Wits Versus Their Genes” [3]. The evolution and rise of antibiotic-resistant microbes prove that bacteria can evolve by genetic adaptation to both antibiotic-enriched and -deprived environments, which drives the evolution of environment-favoured strains by mutation or gene transfer, and, hence, it is up to us to use our wits to keep up with these changes [4].

Antimicrobial peptides (AMPs) evolved through billions of years as part of our innate immune system and are agents produced by various cells in the human body that play an important role in our ability to respond to infections. Lysozyme, the first natural antimicrobial peptide isolated from our body, discovered by Sir Alexander Fleming in 1922, marked the birth of modern innate immunity. Nisin, a polycyclic antibacterial peptide composed of 34 amino acids was discovered in 1928, belonging to the class I bacteriocins produced by *Lactococcus lactis*, was the first AMP to be isolated from a bacterium. Nisin is commonly used as a food preservative because of its inhibitory activity against other food spoilage microorganisms and is used in more than 80 countries (approved by the European Union in 1983 and FDA in 1988). Since then, many antibiotics and antimicrobial peptides have been discovered. Later, in 1939 an antimicrobial agent from a soil strain of *Bacillus* was extracted by Dubos that was found to be effective against mouse pneumococcal infections [5,6]. This was identified in the following year as gramicidin by Hotchkiss and Dubos. Gramicidin showed some toxicity associated with intraperitoneal application, but it was found to be highly effective for the topical treatment of wounds and ulcers, especially during World War II [7,8]. Thaumatin is an AMP derived from the African shrub *Thaumatococcus daniellii* that is a 22 kDa protein that displays antifungal activity. Thionins are small proteins (~5 kDa) that are stabilised by three to four disulfide bonds and display a broad range of toxicity against bacterial, fungal, mammalian, and insect cells [9]. The plant defensin RsAFP2 derived from radish has been shown to offer protection against Candidiasis in a mouse model of infection, whereas other plant defensins have been engineered in transgenic plants that provide resistance against fungal pathogens in crop plants [10,11]. The synthetic AMP **12f** (His(2-biphenyl)-Trp-His(2-biphenyl)) is an amphiphilic peptide of a new structural class that has been shown to have potent fungicidal activity against *Cryptococcus neoformans* with high selectivity [12]. The Antimicrobial Peptide Database (APD) contains 3324 antimicrobial peptides (391 bacteriocins/peptide antibiotics from bacteria, 5 from archaea, 8 from protists, 22 from fungi, 364 from plants, and 2446 from animals, including some synthetic peptides) [13]. AMPs have redefined the way we think about immune defence and human disease and have different names such as host defence peptides, alarmins, and defensins. However, in exploring their potent role in cell behaviour, the term AMP will be used in this review.

## 2. Different Classes of AMPs and Their Need in Modern Medicine

**Figure 1:**
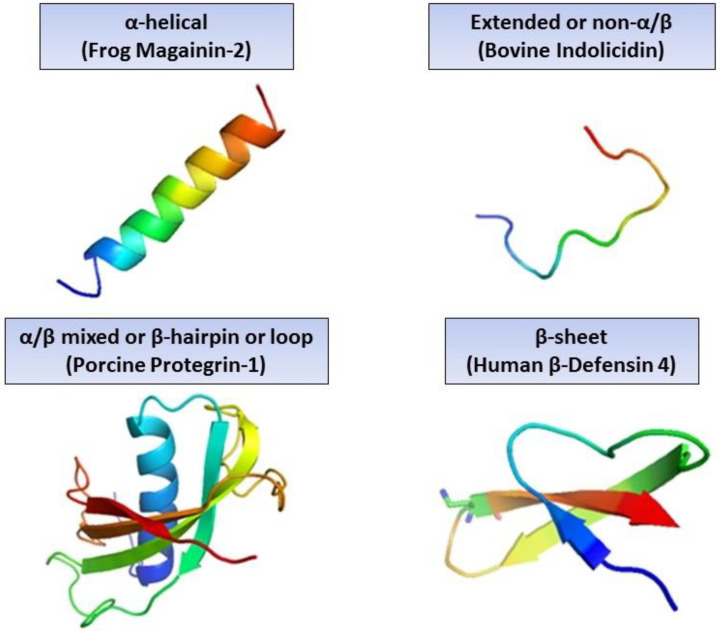
Various secondary structures of antimicrobial peptides.

AMPs, in general, consist of 10–60 amino acid residues, and these peptides lack any specific consensus amino acid sequences that are associated with biological activity. Most of them maintain certain common features, such as containing a +2 to +9 charge (cationic), mainly due to lysine and arginine residues, and are relatively hydrophobic and amphipathic in nature [14]. Based on their amino acid composition, size, and conformational structures, AMPs can be divided into four different categories according to their secondary structure: α-helices, β-sheet, linear extended, non-α/β, α/β, loop, or β-hairpin peptides [15,16]. Of these, α-helical structures and β-sheet peptides are more common [17] (Figure 1).

AMPs with α-helix structures, such as magainin-2 and LL-37, are unstructured in an aqueous solution and assume an amphipathic helical shape upon their interaction with bacterial cell membranes or with organic solvents [18]. β-sheet peptides are cyclic AMPs that are stabilised by disulfide bridges and include protegrins (cathelicidin family); defensins (the largest group of β-sheet AMPs); and tachyplesins (isolated from the horseshoe crab) [19,20]. β-sheet AMPs possess a more stable structure in solution without undergoing any major conformational changes while interacting with a membrane environment [21]. Bactenecin, Protegrin-1, Gramicidin, Thanatin, and lactoferricin B are peptides with a loop structure that is stabilised by disulfide, amide, or isopeptide bonds [17,22]. It is important to note that anionic AMPs also exist, and they also contain several acidic amino acids, such as aspartic and glutamic acid, e.g., dermcidin produced by human sweat glands [23,24,25].

The silent pandemic of antimicrobial resistance is taking its toll, with the misuse of antibiotics in agriculture and animal husbandry impacting the farm to fork transmission of disease affecting people that are in serious need of life changing treatment as a result of a lack of government regulations, particularly in the agri-food sector [26,27]. In 2019 alone, 1.3 million people died because of antibiotic-resistant infections, and by 2050 it is estimated that 10 million people will die as a result, leading to more deaths than cancer and diabetes combined and costing the economy GBP 66 tn [28,29]. This has led to finding alternatives such as phage therapy and the use of nanomaterials/nanoparticles and AMPs for antibiotic resistance and increased biofilm-associated infections [30,31]. However, some mechanisms of AMPs remain incompletely understood, and more work needs to be conducted to determine the efficacy and safety of AMPs and their synergies with lifesaving drugs to recognise their full potential. AMPs can kill a broad range of microbes, including bacteria, fungi, parasites, and viruses [32]. A subset of these peptides and their synthetic derivatives act as potent inhibitors of microbial biofilms, which are associated with the majority (about two-thirds) of all infections in humans, exhibiting increased tolerance to treatment with numerous clinically available antibiotics [33,34].

## 3. Sources of AMPs and Their Inhibitory Effects

AMPs can be divided into four major categories: those derived from mammals (human host defence peptides), amphibians and fish, microorganisms, and insects, according to the APD3 database (Table 1). The AMPs found in oceans have also attracted widespread attention.

### 3.1. Mammalian AMPs

Mammalian antimicrobial peptides are found in human, sheep, cattle, and other vertebrates. Cathelicidins and defensins are the two main families of AMPs [24]. Cathelicidin are polypeptides that are primarily stored in the lysosomes of macrophages and polymorphonuclear leukocytes in humans and are encoded by the CAMP gene, which encodes the peptide precursor CAP-18, which is processed by proteinase 3-mediated extracellular cleavage into the active form LL-37 [63]. It is the only peptide of the cathelicidin family found in the human body [64].

The first reported AMP of animal origin was defensin, which was isolated from rabbit and guinea pig granulocytes in 1956 [65,66]. Defensins can be divided into α-, β-, and θ-defensins depending on the positions of disulfide bonds (Reddy et al., 2004). α-defensins are small peptides with 29–35 amino acid residues and three intramolecular disulfide linkages. Humans are known to possess six α-defensins, four human neutrophil peptides (HNP 1–4) expressed by granulocytes and certain lymphocytes, and two (HD-5 and 6) expressed by intestinal Paneth cells [35].

**Figure 2:**
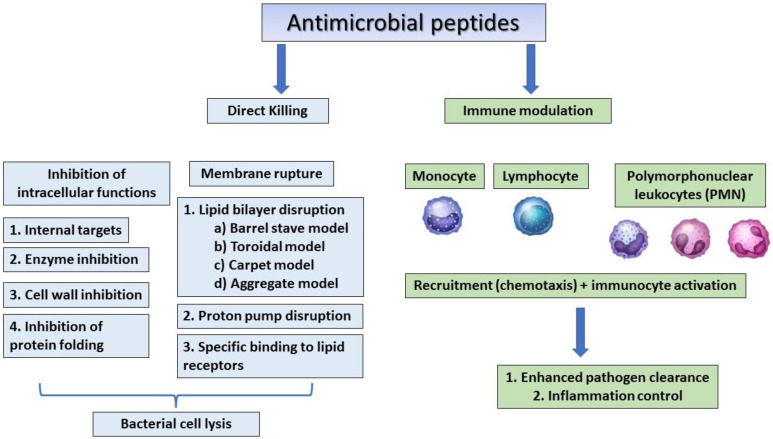
The general mechanism of action of antimicrobial peptides.

The human β-defensins are of four types (HBD 1–4) and are composed of up to 45 residues that are expressed by the epithelial skin cells, and psoriatic scales and the synthesis of these peptides can be induced by cytokines such as TNF- α and IL-1 β [36,37]. Human host defensins can protect humans from microbial infections but show different levels of expression at every stage of human growth. For example, cathelicidin LL-37, is usually detected in the skin of new-borns, whereas human beta-defensin 2 (HBD-2) is often expressed in the elderly [67]. Human defensin peptides are present in several parts of the body, including the skin, eyes, ears, mouth, respiratory tract, lung, intestine, urethra, tracheal epithelium, and at elevated levels (HBD-2 in particular) in human keratinocytes exposed to pathogenic bacteria [24,68]. AMPs from human breast milk can play an important role in decreasing the morbidity and mortality of breast-feeding infants [69]. A recent study showed that the levels of AMPs were similar in the breast-milk-fed preterm infants with and without late-onset neonatal sepsis, but since preterm infants with late-onset neonatal sepsis consumed significantly less breast milk, the levels of milk AMPs were low. This indicates that the concentrations of lactoferrin and defensins in preterm breast milk have antimicrobial activity against neonatal pathogens [69]. Dairy is an important source of AMPs, which are produced by the enzymatic hydrolysis of milk, of which lactoferricin B (LfcinB) is well-known for its antimicrobial properties that affect intracellular activities, leading to bacterial inhibition [38]. In addition to antimicrobial activity, cathelicidins and defensins have also been shown to affect immune regulation, apoptosis, and wound healing [39]. The mechanisms of action of antimicrobial peptides are summarised in Figure 2.

### 3.2. Amphibian and Fish AMPs

Antimicrobial peptides from amphibians play an important role in the protection of amphibian species from pathogens and global decline [70,71]. Frogs and toads are considered the main and the largest source of amphibian AMPs. Cancrin was the first AMP to be discovered from the sea amphibian *Rana cancrivora*, a crab-eating frog [72]. The most important being the magainins, which are peptides constitutionally produced by mucous glands in the skin of several frogs of genera such as *Xenopus*, *Silurana*, *Hymenochirus*, and *Pseudhymenochirus* possessing antibacterial, antifungal, and antitumor properties. Others include peptides such as bombinins, cathelicidin, maximins, phylloxin, plasticins, and palustrin, which are antibacterial; buforin, esculentins, and pseudins, which are antibacterial and antifungal; dermaseptins that are antiviral, antibacterial, antifungal, and anticancer; fallaxin, which is antibacterial; and leishmanicidal and ranateurins, which show antibacterial and anticancer properties [40]. Cathelicidins, defensins, hepicidins, piscidins, and histone-derived peptides are AMPs found in fish and also occur in humans, cattle, pigs, and horses [73]. The broad-spectrum antimicrobial peptides (HFIAP-1, -2, and -3) were the first cathelicidins that were isolated from the intestinal tissues of *Myxine glutinosa* (Atlantic hagfish), which possessed broad-spectrum antimicrobial activity against a number of Gram-positive and -negative bacteria [41]. The cathelicidin codCATH, isolated from Atlantic Cod (*Gadus morhua*), which is rich in glycine residues, shows activity against Gram-negative bacteria, whereas rtCATH1 (R146-P181) and rtCATH2 (R143-I178), isolated from Rainbow Trout, are active against *Lactococcus garvieae* and other Gram-negative fish pathogens [42,43]. Fish defensins/defensin-like proteins consisting of six conserved cysteine motifs were initially discovered in zebrafish, Fugu, and tetraodon using a database-mining approach [74]. These show potent activity against bacteria and fish-specific viruses, e.g., cod defensin defb, identified in Atlantic cod, shows antibacterial activity against the Gram-positive bacteria *Planococcus citreus* and *Micrococcus luteus* [44], whereas EcDefensin, isolated from the orange-spotted grouper, *Epinephelus coioides*, is shown to inhibit the replication of a DNA virus, Singapore Grouper Iridovirus (SGIV), and an RNA marine fish virus, viral nervous necrosis virus (VNNV) [75]. Fish hepcidins are rich in cysteine and are hormones involved in iron regulation that share a β-sheet-composed hairpin structure linked via four disulfide bonds, e.g., Om-hep1 from *Oryzias melastigmus* is active against Gram-positive bacteria such as *C. glutamicum* and *S. aureus* and Gram-negative pathogens such as *E. coli* MC1061, *A. hydrophila*, and *Pseudomonas stutzeri* [76]. Piscidins share an α-helical structure similar to magainins and are linear AMPs, which are amphipathic in nature [77]. Pleurocidin is a magainin isolated from winter flounder (*Pleuronectes americanus*) and is active against both Gram-positive and -negative pathogens [45].

### 3.3. AMPs from Microorganisms

#### 3.3.1. AMPs from Gram-Positive Bacteria

Antimicrobial peptides such as nisin and gramicidin are produced by Gram-positive bacteria such as *Lactococcus lactis* and *Bacillus brevis* [78]. Both ribosomally and non-ribosomally synthesised AMPs have been found in Gram-positive bacteria [46].

Ribosomally synthesised bacterial AMPs are termed as bacteriocins [79]. There are four classes of bacteriocins produced by Gram-positive bacteria: (I) lantibiotics, (II) non-lantiboitics, (III) large-sized bacteriocins, and (IV) uniquely structured bacteriocins [78]. Small peptides (<5KDa, 19–38 amino acids), which are post-translationally modified and contain unusual amino acids, belong to the class of lantibiotics. Subclass 1a is positively charged and acts by forming pores (e.g., nisin, epidermin, gallidermin, and Pep5). Subclass 1b is negatively charged, globular, inflexible, and acts by inhibiting crucial enzymes in targeted pathogens (e.g., mersacidin, actagardine, and cinnamycin) [80]. Non-lantibiotics are small, heat-stable peptides with limited post-translational modification. They act by increasing cell permeability by pore formation. Subclass IIa are disulfide-containing linear AMPs (e.g., pediocin PA-1 and enterocin P) [81,82], subclass IIb contain α and β subunits (e.g., plantaricin EF, lactococcin G, thermophilin 13, and lactacin F) [83], subclass IIc are small cyclic peptides with covalently linked N and C-termini (e.g., enterocin AS-48, gassericin A, circularin A, and lactocyclicin Q) [84], and subclass IId remain to be characterised (e.g., lactococcin A, B, and 972 and enterocinL50) [83]. Large-sized bacteriocins (>30 kDa) are also called bacteriolysins and are heat-labile peptides (e.g., zoocin A, lysostaphin, and enterolysin A) [85]. Uniquely structured bacteriocins contain amino acids, lipids, or carbohydrates and are susceptible to lipolytic or glycolytic enzymes (e.g., plantaricin S, leuconocin S, and lactocin 27) [86,87].

Non-ribosomally synthesised AMPs are cyclic heptapeptides consisting of a tripeptide side chain linked to an N-terminal fatty acyl tail and are obtained from the Gram-positive spore-forming soil bacterium *Paenibacillus polymyxa* [47,48]. So far, at least 10 different groups of polymyxin lipopeptides have been identified; they are polymyxins A, B, C, D, E, F, M, P, S, and T, with polymyxin B and E (colistin) being used clinically, and are active against critically important pathogens listed by the WHO such as *A. baumannii*, *P. aeruginosa*, and *S. maltophilia* [48]. Tridecaptins (A, B, and C) are a re-emerging class of non-ribosomal antibacterial peptides (NRAPs) that are isolated from *Paenibacillus polymyxa* AR-110, B-2, and E-23, respectively, and show potent activity against drug-resistant strains of Gram-negative pathogens, where they selectively bind to the Gram-negative analogue of peptidoglycan precursor lipid II on the outer leaflet of the inner membrane, disrupting the proton motive force [88].

#### 3.3.2. AMPs from Gram-Negative Bacteria

Bacteriocins are AMPs that are isolated from Gram-negative bacteria such as *E. coli*, *Klebsiella*, and *Pseudomonas* spp. [49] that show a narrow-spectrum activity against Gram-negative pathogens. They are classified into four different classes: colicins, colicin-like bacteriocins, microcins, and phage tail-like bacteriocins.

Colicins (>10 kDa) are plasmid-encoded AMPs produced by *E. coli* and specifically bind to cell surface receptors before translocation through the outer membrane, periplasm, and inner membrane into the cell cytoplasm [50]. Colicin A, K, and U bind to BtuB, Tsx, and OmpA receptors, respectively [89]. Colicins are divided into three classes according to their mechanisms of action: (i) forming channels or pores in the cytoplasmic membrane (e.g., colicin A, B, and E1), (ii) DNA degrading (e.g., colicin E2, E7, E8, and E9), targeting rRNA (e.g., ColE3, ColE4, ColE6, and DF13) or tRNA (e.g., ColE5, ColE6, and Col D), and (iii) inhibition of murein and lipopolysaccharide synthesis (e.g., colicin M) [51,52].

Colicin-like bacteriocins are produced by Gram-negative bacteria such as *P. aeruginosa* (e.g., S-type pyocins) and *Klebsiella* spp. (klebicins) and are structurally and functionally similar to *E. coli* colicins [90,91]. S-type pyocins (AP41, S1–S5) are sensitive to proteases and induce cell death by cleaving DNA (AP41 and S1–S3) or RNA (S4) or via pore formation (S5) [92]. The action of klebicins occurs via endonuclease activity, pore formation, and/or by the degradation of peptidoglycan [91].

Microcins are small peptides (<10 kDa) produced by bacteria belonging to *Enterobacteriaceae* and are classified into two subclasses (e.g., Colicin V and Microcin C7) [50]. Subclass I (<5 kDa) undergo extensive post-translational modification compared to subclass II (>5–10 kDa), which are unmodified or slightly modified post-translationally [40].

High molecular weight cylindrical peptides with high similarity to the phage tail structure are referred to as phage tail-like bacteriocins [40]. These are also divided into two subclasses. Subclass I consists of R-type bacteriocin related to the contractile tail of phages belonging to the family Myoviridae. Subclass II consists of F-type bacteriocins related to *Siphoviridae* phage tails. R- and F-type pyocins are produced by *Pseudomonas aeruginosa* and are encoded in a gene cluster comprising a DNA region greater than 40 kb [50].

#### 3.3.3. Fungal AMPs

Many fungal AMPs not only show inhibitory activity against common pathogenic fungi, such as *Aspergillus* and *Candida* spp. in humans, but also against yeast and filamentous fungi (e.g., *Aspergillus flavus*), which affect food and agriculture [24]. Fungal AMPs are divided into peptaibols (found in *Trichoderma* spp.) and defensins (found in *Pseudoplectania*, *Coprinopsis*, and *Microsporum* spp.) [40]. The fungal AMPs contain 5–21 amino acids, with a high proportion of non-proteinogenic amino acids, such as α-aminoisobutyric acid, and typically have an acylated N-terminal residue and an amino alcohol, such as phenylalaninol or leucenol, attached to the C-terminal [93]. Alamethicin is the most widely studied peptaibol isolated from *T. viridea*, which is active against both Gram-positive bacteria such as *E. faecalis*, *S. hemolyticus*, and *S. aureus* and Gram-negative bacteria such as *E. coli*, *K. pneumoniae*, and *P. aeruginosa*, with antimicrobial activity against fungi as well [93,94,95]. Peptaibols can be classified as short-chain, consisting of 5–10 amino acids; medium-chain, with 11–16 amino acids; and long-chain, with 17–21 amino acids, with the primary mechanism of action involving membrane disruption [53]. Peptaibols of ≥15 amino acids can form helical structures that oligomerise, forming ion channels in the membrane [96]. Peptaibols of <15 amino acids act by a combination of membrane disruption (e.g., the formation of transmembrane channels or by a barrel-stave mechanism) and also affect different molecular targets.

The fungal defensin-like peptides are cysteine-rich peptides that show high sequence and structural similarities to defensins from microorganisms, plants, and animals [74,97]. Plectasin isolated from *Pseudoplectania nigrella* was the first fungal defensin to be characterised, which showed inhibitory activity against Gram-positive bacteria such as *S. pyogenes*, *C. diphtheriae*, and *S. aureus* [54]. Micasin from *Microsporum canis* was found to show broad-spectrum antibacterial activity against both *P. aeruginosa* and methicillin-resistant *S. aureus* by affecting protein folding [55].

#### 3.3.4. Viral and Bacteriophage AMPs

Antiviral drug resistance is an increasing concern in immunocompromised patients, with the limited efficiency of commonly used antiviral drugs making viral AMPs ideal candidates as potential therapeutic agents [98]. Antiviral agents act at different levels, such as inhibiting the activity of viral reverse transcriptase, retroviral integrase, or proteases or the inhibition of the transport of circular viral DNA to the nucleus, impairing cellular processes [99]. Antiviral AMPs integrate into viral envelopes, causing membrane instability and thereby preventing the viruses from infecting host cells [100]. Melittin (the main compound found in the venom of the European honeybee *Apis mellifera*), which also has anticancer activity, has an inhibitory activity against both enveloped and non-enveloped viruses, including coxsackievirus, enterovirus, influenza A viruses, human immunodeficiency virus (HIV), herpes simplex virus (HSV), Junín virus (JV), respiratory syncytial virus (RSV), vesicular stomatitis virus (VSV), and tobacco mosaic virus (TMV) [56]. It has also been suggested that melittin impedes cell fusion in HSV-1 glycoprotein K mutants by interfering with the activity of sodium potassium ATPase, an essential enzyme involved in the membrane fusion process [101]. Antiviral AMPs can also prevent viral particles from entering the host cells by binding to specific mammalian cell receptors, e.g., lactoferrin inhibits HSV infections by binding to heparan sulfate proteoglycans, which play an important role in the attachment of HSV viral particles to the host cell surface, thereby blocking any interactions with the virus receptor [102].

Bacteriophages are a type of viruses that infect bacteria, and the word “bacteriophage” literally refers to “bacteria eating”. These viruses multiply in the presence of bacteria and kill them to release their progeny. This mechanism is used in clinical treatment to kill the target bacteria, and many studies are ongoing to develop phages into a more potent alternative. In vitro and in vivo works have shown that phages have a high efficacy in killing multidrug-resistant bacteria [103]. Whole phage-based treatments are time-consuming; thus, phage-derived antimicrobial peptides such as endolysins are being studied as an alternative medicine to treat bacterial infections.

Many phage proteins, including endolysins, virion-associated peptidoglycan hydrolases (VAPGHs), depolymerases, and holins, display antibacterial activity [104,105]. Phage AMPs are of two types, phage-encoded bacteriolytic proteins produced at the end of the lytic cycle and phage-tail complexes [104,106]. Based on the enzymatic activity, endolysins can be divided into four major types, namely acetylmuramidases, glucosaminidases, amidases, transglycosylases, and endopeptidases [107]. Each endolysin is known to have a specific enzymatic function. The endolysin structure dictates the function and activity of the endolysin. In general, endolysin is made up of two active sites, one at the N-terminal linked by a short flanking region to the cell-wall-binding domain at the C-terminal [108]. The endolysin specific to the Gram-positive bacteria are known to resemble fungal cellulases, and Gram-negative-specific endolysins have multiple globular structures that are drawn from the primary endolysin motifs [109].

Phage lysins are peptidoglycan-hydrolysing enzymes ranging from 25 to 40 kDa. They act by weakening the peptidoglycan bacterial cell wall, creating holes that permit phage progeny to exit the cell, resulting in rapid bactericidal activity. They may also possess synergistic activity with cell-wall-inhibiting antibiotics, antibiofilm activity, and are heat-stable up to a temperature of 50 °C [57,58]. Examples include LysAB2 P3, which shows inhibitory activity against *Acinetobacter baumannii* [110], and PlyV12, a phage lysin that exhibits broad bactericidal activity against enterococci and other Gram-positive pathogens such as *S. pyogenes* and *S. aureus* [111]. VAPGHs are genus/species-specific lytic enzymes that are encoded by double-stranded DNA phages that specifically degrade peptidoglycan. They have a C-terminal cell-wall-binding domain and one or more N-terminal catalytic domains [112]. VAPGHs can be classified into three categories based on the peptidoglycan cleavage site, namely, glycosidases (muramidases that cleave between N-acetylmuramic acid and N-acetylglucosamine residues, similar to lysozyme), amidases (those that cleave between N-acetylmuramic acid and the first highly conserved L-alanine residue), and endopeptidases (cleave between two amino acid residues) [113]. VAPGHs are active against both Gram-positive and -negative bacteria. VAPGH HydH5 of Φ vB_SauS-phiIPLA88 is active against *S. aureus*, whereas Phage Φ6 contains protein P5, an endopeptidase that shows inhibitory activity against *P. aeruginosa* [112,114]. Phage polysaccharide depolymerases are enzymes that degrade the macromolecule carbohydrates of the bacterial cell wall [115]. *Klebsiella* phage ΦK64-1 encodes several depolymerases that show activity against a number of *Klebsiella* capsular polysaccharides, whereas *P. putida* phage AF degrades the extracellular polysaccharides involved in the formation of the biofilm matrix of *P. putida* [116,117,118]. Hence, depolymerases may be effective against bacteria producing biofilm such as *P. mirabilis*, *E. coli*, *S. suis*, *K. pneumoniae*, and *P. aeruginosa* and in the treatment of biofilms formed by foodborne bacteria, such as *Campylobacter* and *Salmonella* [119,120,121,122,123]. OmniLytics Inc. (Sandy, UT, USA), has developed two products, BacWash^TM^ for *Salmonella* and Finalyse^TM^ for *E. coli* O157:H7, marketed by Elanco (Greenfield, IN, USA). Intralytix Inc. (Baltimore, MD, USA) developed three phage products, ListShield^TM^, EcoShield^TM^, and SalmoFresh^TM^, to be used in the food industry against *L. monocytogenes*, *E. coli*, and *Salmonella*, respectively, which can help in making food more secure, thereby protecting public health [123]. Holins are a diverse group of small hydrophobic proteins produced by dsDNA bacteriophages (<150 amino acids) that are involved in the regulation of bacterial lysis time by guiding the phage muramidases to the peptidoglycan layer [59]. Canonical holins form large pores on one side of the bacteria, locally exposing the peptidoglycans to cytoplasmic canonical endolysin molecules [124]. Pinholins form small pores that result in membrane depolarisation, triggering signal-arrest-release (SAR) endolysin activation and the degradation of peptidoglycans in the whole cellular periplasmic space [124]. The *S. aureus* bacteriophage GH15 produces the holin HolGH15, which has broad antibacterial activity against the foodborne pathogen *L. monocytogenes* [60]. Phage-derived endolysins are a powerful weapon for combatting antibiotic resistance, as they exhibit rapid activity against bacterial cells, and their remarkable ability to lyse bacterial cells, even in the absence of bacterial multiplication, makes them a superior medicine over whole phage therapy.

#### 3.3.5. Insect AMPs

Antimicrobial peptides are mainly synthesized in the fat bodies and blood cells of insects and are a source of strong adaptability and survival [125]. Insect AMPs exhibit an antimicrobial effect by disrupting the cell membrane and prevent microbes from developing drug resistance [126]. Cecropin A, found in cecropia moth (*Hyalophora cecropia*) and bees, shows activity against different inflammatory diseases and cancers [61]. Insect AMPs such as cecropins, ponericins, defensins, lebocins, drosocin, metchnikowin, gloverins, diptericins, and attacins are a heterogeneous group of immunity-related proteins that exhibit an antimicrobial effect, mainly against Gram-negative bacteria [127]. A peptide derived from the royal jelly of honeybees, Jellein, shows promising effects on several bacteria and fungi, and its conjugated form with lauric acid can inhibit the parasite *Leishmania major* [62].

## 4. Bacterial Biofilm Formation in Priority Pathogens

A biofilm is an assemblage of one or more types of microorganisms that can grow on many different surfaces and is irreversibly associated (not removed by gentle rinsing) with a surface and enclosed in a matrix of primarily polysaccharide material [128]. Bacterial biofilms appear in both mono- and multilayers, depending on the attachment of the exopolysaccharide (EPS) matrix and the involvement of neighbouring bacteria [129]. Due to their hydrophobic nature, dissolved organic molecules accumulate on the solid–water interface, resulting in a film, and then they form small groups of bacteria, known as micro-colonies. EPSs such as proteins, glycopeptides, glycolipids, lipopolysaccharides, and extracellular DNA accumulate in the attachment [130]. When the second stage is complete, adhesion becomes irreversible in the absence of physical or chemical intervention, leading to the formation of a mature biofilm with micro-colonies assuming a distinct phenotype with a different gene expression than their planktonic counterparts. The differentiation of cells is activated by the deposition of N-acyl homoserine lactones, a sensing molecule involved in cell-to-cell communication, which is also known as quorum sensing [131]. Biofilm formation is an excellent survival strategy in a nutrient-poor environment and is favoured under starved conditions. Besides this, higher antibiotic resistance is observed in biofilm because bacteria that are starved of nutrients adapt to combat aggressive environmental challenges [129,132]. Antibiotic resistance mechanisms, such as the occurrence of mutations and efflux pumps, are better understood in the planktonic form compared to biofilm [133].

The “ESKAPE” group of pathogens have garnered particular attention in recent years for their ability to escape or evade common therapies through antimicrobial resistance mechanisms and biofilm formation. They were first described in 2008, consisting of *Enterococcus faecium*, *Staphylococcus aureus*, *Klebsiella pneumoniae*, *Acinetobacter baumannii*, *Pseudomonas aeruginosa*, and *Enterobacter* spp. [134]. Of these, carbapenem-resistant *Acinetobacter* and *Enterobacteriaceae* are in the CDC’s 2019 top five list of antibiotic-resistant bacteria that are considered as urgent threats. Besides this, clarithromycin-resistant *Helicobacter pylori*, the causative agent of gastritis, gastric and duodenal ulcers, mucosal-associated lymphoid tissue, and gastric cancer, is regarded as a high-priority bacterium by the World Health Organization (WHO) [27,135]. Fluoroquinolone resistance in *Campylobacter jejuni* is another high priority for antibiotic research and development by the WHO, as *Campylobacter* is the number one cause of bacterial gastrointestinal disease in the United Kingdom, costing the economy GBP 1 billion each year [4,135]. The understanding of the biofilm drug-resistance mechanisms is crucial for developing effective antimicrobial treatment, especially in critically ill patients. The therapeutic potential of antimicrobial peptides lies in their ability to kill bacteria effectively without exhibiting significant cytotoxicity toward mammalian cells. Additionally, there is also synergy observed between insect peptides and conventional antibiotics to combat antibiotic-resistant pathogens [129]. A list of antimicrobial peptides that are effective against different pathogens is summarised in Table 2.

### 4.1. Enterococcus *spp.*

*Enterococcus faecium* is a Gram-positive coccus, commonly involved in hospital-acquired infections in immunocompromised patients. Vancomycin-resistant enterococci (VRE) strains, particularly vancomycin-A-resistant *E. faecium* have risen in recent years, with some forming thicker biofilms that act as mechanical and biochemical shields that protect the bacteria from the antibiotics, allowing them to grow in a variety of environments, such as urinary catheters and prosthetic heart valves in the body [171,172]. The AMP C16-KGGK was shown to be most effective against *E. faecalis* grown in suspension and in biofilms, followed by C16-KGGK, which was formulated with one of two polymers, poly (lactic acid co castor oil) (DLLA) or ricinoleic-acid-based poly (ester-anhydride) P(SA-RA) [136]. *Enterococcus faecalis* is a common cause of biofilm-associated opportunistic infections and biofilms formed on the dentinal walls of the root canal and is frequently the cause of endodontic treatment failure and secondary apical periodontitis [137]. The antifungal peptides KP and L18R have been shown to have antibacterial activity against planktonic *E. faecalis* cells at micromolar concentrations with a promising potential of L18R for the development of new strategies for endodontic infection control [137]. Buwchitin, a ruminal peptide, at a minimum inhibitory concentration (MIC) was bacteriostatic against *E. faecalis* cells and inhibited their growth in vitro by 70% compared to untreated cells, suggesting that buwchitin plays a role in antimicrobial activity against *E. faecalis* [138]. It was also recently shown that bulky non-natural amino-acid-substituted P-113 derivatives have strong microbicidal and synergistic activities with vancomycin and were shown to be effective against vancomycin-resistant *E. faecium*, *S. aureus*, and *E. coli*. These AMPs also reduced the release of antibiotic-induced lipopolysaccharide (LPS) from Gram-negative bacteria, with Bip-P-113 peptide demonstrating the best inhibitory activity, antibiotic synergism, and supernatant LPS-neutralizing activities against various microbes [139].

### 4.2. Staphylococcus aureus

*Staphylococcus aureus*, a Gram-positive coccus occurring in grape-like clusters, is an unharmful bacterium that is a part of the normal human skin microbiota. However, it has the ability to cause infections when it enters parts of the body that it does not normally inhabit, such as wounds or soft tissues. Infections caused by this pathogen are common both in community-acquired and hospital-acquired settings, and due to the emergence of multidrug-resistant strains such as MRSA (methicillin-resistant *Staphylococcus aureus*) treatment remains challenging [173].

The antimicrobial peptide NA-CATH: ATRA1-ATRA1, a synthetic helical cathelicidin identified in reptiles such as the Chinese cobra, *Naja atra* (NA-CATH), inhibited *S. aureus* biofilm formation in the presence of salt, exhibiting antibiofilm activity at lower peptide concentrations than NA-CATH, LL-37, and D-LL-37 and has low cytoxicity against host cells but did not affect bacterial attachment [141]. The glycopeptide resistance in biofilms formed by *S. aureus* is regulated by the glycopeptide resistance-associated two-component system, GraRS. It has been shown earlier that graRS single mutants are resistant to the muramidase activity of lysozyme but are sensitive to cationic antimicrobial peptides, including the human lysozyme-derived peptide _107_R-A-W-V-A-W-R-N-R_115_ (LP9), polymyxin B, or gallidermin, indicating the potential of AMPs for treating infections [142]. Phage-derived bacteriocins (endolysins) are produced by matured phage virions, which can also be used for treatment, and one such example is Staphefekt™ SA.100™ (Micreos, Bilthoven, The Netherlands), constructed by Fritz Eicheseher and Martin Loessner at ETH Zurich (Switzerland). This is the first endolysin for human use that only targets *Staphylococcus aureus* [143,144]. In 2013, endolysin SAL-1 became the first antimicrobial peptide to be approved for a clinical trial (phase I) as a therapy for staphylococcal infections [174].

### 4.3. Klebsiella pneumoniae

*Klebsiella pneumoniae* is a Gram-negative non-motile bacterium with a capsule that is found in the environment and is often associated with pneumonia in patients with alcohol use disorders or type II diabetes. It can colonise the human mucosal surfaces of the oropharynx and gastrointestinal tract, can display high degrees of virulence and antibiotic resistance, and is considered the most common cause of hospital-acquired pneumonia in the United States [175].

The extracellular polysaccharide capsule of Klebsiella pneumoniae resists penetration by antimicrobials and helps it evade the human innate immune system. A 17-amino-acid antimicrobial peptide, PepC, with bactericidal activity against *Escherichia coli* and *Acinetobacter baumannii* strains was identified, but it had little activity against clinical multidrug-resistant and hypermucoviscous *K. pneumoniae* [145]. On modifying PepC, its analogs A6, A12, and A19 had the lowest overall MICs toward *E. coli* and *K. pneumoniae*, indicating that active antimicrobial peptides could be derived from inactive parent sequences [145]. On the contrary, the interaction of two AMPs, BMAP-27 and Bac71–35, with the biofilms of the *K. pneumoniae* strains KpTs101, KpTs113, and KpMn7 was shown to lead to the formation of complexes, to some extent preventing the antimicrobials from reaching the bacterial membrane and preventing a proper interaction with it, therefore lowering the activity of the AMPs [176]. Perhaps targeting the outer membrane protein OmpA, which is responsible for membrane integrity, the transport of molecules, and pathogenesis, might be a useful approach, as *K. pneumoniae* ompA mutants are more susceptible to AMPs than the wild type [177]. Hence, it is important to fully understand the role and function of specific AMPs in the prevention of biofilms. The cathelicidin-derived peptide D-11 was shown to sensitize *K. pneumoniae* to a range of antibiotics in vitro, ex vivo, and in vivo, displaying strong synergy with several antibiotics, and is potentially better than some outer membrane-active peptides that are already in clinical trials [146]. Clinically relevant combinations of AMP DJK-6 and meropenem have been shown to prevent the planktonic growth and biofilm formation of *K. pneumoniae* strain1825971 [147]. DJK-6 was able to enhance the ability of meropenem by at least 16-fold and eradicate biofilms formed by this strain. Hence, the potentiation of meropenem by DJK-6 and synergies of AMPs with antibiotics represent a promising strategy in treating infections caused by *Klebsiella* spp. [147]. AMP IDR-1018 has also been shown to exhibit broad-spectrum antibiofilm activity against a variety of hospital pathogens, including *K. pneumonia* and *Pseudomonas aeruginosa* [148]. The high effectiveness of the AMPs citropin 1.1 and CAMEL against the planktonic forms of microbes was shown earlier, resulting in the possibility of using these compounds in preventive management [149].

### 4.4. Acinetobacter baumannii

*Acinetobacter* spp. are a group of Gram-negative bacteria that are readily found throughout the environment, including drinking and surface waters, soil, sewage, and various types of foods. *Acinetobacter baumannii* has gained a lot of interest in the past decade due to its increasing prevalence in the hospital environment in addition to its ability to resist antimicrobial compounds and is identified as a major threat, especially in immunocompromised patients [178]. Carbapenem-resistant *A. baumannii* is also a critical priority pathogen listed by the World Health Organization.

The antimicrobial peptide Cec4 was shown to have a strong effect on carbapenem-resistant *A. baumannii*, and a comparative transcriptome analysis shows that multiple metabolic pathways, two-component regulation systems, quorum sensing, and antibiotic synthesis-related pathways in *A. baumannii* biofilms are affected after Cec4 treatment, representing a new choice for the prevention and treatment of clinical infections [150]. Besides this, the MIC of the human-derived cationic peptide LL-37 and its truncated fragments against drug-resistant *A. baumannii* were 16–32 μg/mL, and it was also shown to inhibit biofilm formation [151]. AMPs, SMAP-29, and TP4 were shown to have prophylactic effects preventing the deaths of mice with pneumonia caused by *A. baumannii*. Two TP4 derivatives (dN4 and dC4) were shown to have therapeutic activity in pneumonia mouse models by peritoneal or intravenous administration, with both inhibiting *A. baumannii* biofilms at higher doses. This suggests that the AMP derivatives dN4 and dC4 represent an excellent treatment strategy for *A. baumannii*-induced pneumonia [152]. The synergistic effects of the AMP melittin were observed with doripenem against *A. baumannii* as well as against *P. aeruginosa*, and the melittin-derived peptides MDP1 and MDP2 demonstrated potent antibacterial activity against MDR *S. aureus* and *E. coli* [179,180]. The enzymatic activity of phage endolysin provides them with narrow- and broad-spectrum activities against the host. Some good examples are plyAB1, a globular endolysin with a glycoside hydrolase activity that is known to be specific against pan-drug-resistant *Acinetobacter baumannii* [153], and ply6A3, a muramidase-type that is known to have a broad host spectrum activity against *Acinetobacter baumannii*, *E. coli*, *Staphylococcus aureus*, *Klebsiella pneumonia*, and *Enterococcus faecalis* [154]. The endolysin plyF307 was shown to significantly reduce planktonic and biofilm-forming cells in *A. baumannii* both in vitro and in vivo, rescuing mice from lethal bacteremia. It is the first highly active therapeutic lysin specific for Gram-negative organisms isolated from the *Acinetobacter* phage, which targets multidrug-resistant *Acinetobacter baumannii* [155].

### 4.5. Pseudomonas aeruginosa

*Pseudomonas aeruginosa* is a critical priority opportunistic pathogen designated by the WHO that affects immunocompromised patients. It is the leading cause of morbidity and mortality in cystic fibrosis (CF) patients and one of the leading causes of nosocomial infections [181].

The peptide LL-37 has been shown to regulate *P. aeruginosa* biofilm formation when used at levels below MIC [156]. AMPs inhibit the protein expression of genes involved in the formation of biofilm in *P. aeruginosa*, which are associated with type IV pili, rhamnolipid synthesis, quorum sensing, and flagella assembly. First, LL-37 significantly reduces the initial attachment of *P. aeruginosa* to the surface, leading to a decrease in the number of bacteria involved in the initial steps of biofilm development. Second, LL-37 promotes a twitching surface motility mediated by the type IV pili by stimulating the expression of genes related to type IV pilus biosynthesis and function. This leads to increased surface motility, causing bacteria to wander across the surface instead of forming biofilms, resulting in thinner and flatter layer formation of biofilms [156]. *P. aeruginosa* and *Staphylococcus aureus*, which are both involved in CF lung infections, possess proteinases that are able to degrade and inactivate LL-37 and lactoferrin, even during lung infections, but LL-37 is also a key factor in the mucosal immunity of the urinary tract [182,183]. Hence, it could offer protection against urinary tract infections [156]. Lactoferrin exhibits specific antimicrobial properties by binding and chelating iron and inhibiting biofilm formation in *P. aeruginosa* [129]. The binding of AMPs to extracellular DNA can also improve the detection of biofilms [184]. Endolysins are also known to have a drastic effect in killing bacterial biofilms. The two most significant biofilm-associated pathogens, *P. aeruginosa* and *Acinetobacter baumannii*, are considered major opportunistic pathogens in severe burn wounds. The endolysin LysPA26 was shown to have a greater efficiency against the biofilm cells of MDR *P. aeruginosa* without pretreatment with an outer membrane permeabilizer [157].

### 4.6. Enterobacter *spp.*

The *Enterobacter cloacae* complex (ECC) consists of closely related bacteria associated with the human microbiota that are increasingly isolated from healthcare-associated infections and are emerging nosocomial pathogens within the Enterobacteriaceae family [185].

Both BMAP-27B and SMAP-29D cathelicidin AMPs were shown to affect the formation of *E. cloacae* bacterial colonies and cause a rapid reduction in the number of colony forming units (CFUs), with BMAP-27B being more bactericidal than SMAP-29D [158]. The AMP LL-37 derivative SAAP-148, through an amino acid substitution of the C-terminal chain of LL-37, shows potent microbicidal activity against several ESKAPE pathogens such as *E. faecium*, *S. aureus*, *K. pneumoniae*, *A. baumannii*, *P. aeruginosa*, and *Enterobacter* spp. without the selection of resistance. SAAP-148 also completely eradicated acute and established biofilm-associated infections in wound infection models associated with methicillin-resistant *S. aureus* and multidrug-resistant Acinetobacter baumannii [140]. The combination of AMP novicidin and rifampin showed synergy in >70% of *Klebsiella*, *Enterobacter*, and *Serratia* group of microbes, with novicidin being able to revive the activity of rifampin by reducing its MIC by 2- to 512-fold [186]. In the same study, a combination of novicidin and ceftriaxone or ceftazidime was synergistic against 89.7% of the ceftriaxone-resistant strains and 94.1% of the ceftazidime-resistant strains [186].

### 4.7. Helicobacter pylori

*Helicobacter pylori* is a Gram-negative spiral bacterium that grows in the digestive tract, attacking the stomach lining. Around 50% of people worldwide have an *H. pylori* infection, and the emergence of multidrug-resistant *H. pylori* poses a public healthcare threat, particularly in low- and middle-income countries, with the World Health Organization classifying clarithromycin-resistant *H. pylori* as high-priority pathogen [27].

The cathelicidin mCRAMP (cathelicidin-related anti-microbial protein)-encoded *Lactococcus lactis* is upregulated in gastric secretion and epithelium inflamed by *H. pylori* infection. In vitro, experiments show that cathelicidin is bactericidal in several *H. pylori* strains such as SD4, SD14, and Sydney strain 1 (SS1) [159]. The human and mouse cathelicidin mCRAMP-encoded *L. lactis* (a probiotic combined host defence peptide) was shown to secrete mCRAMP both in vitro and in vivo. This *L. lactis* strain was able to prevent acute ulcerative colitis through multiple actions in mice, offering protection against both the non-drug-resistant *H. pylori* strain SS1 and the clarithromycin-resistant *H. pylori* strain 10783 [160]. Cathelicidin plays an important role in the gastric mucosa, leading to the eradication of *H. pylori* infection by modulating the inflammatory responses and carcinogenesis in the stomach and may assist in tissue repair through specific actions in the gastric epithelium [161]. Tilapia Piscidin 4 (TP4) was shown to inhibit the growth of both antibiotic-sensitive and -resistant *H. pylori* via membrane micelle formation, leading to membrane depolarisation and extravasation of the cellular constituents and is proposed to be an effective and safe monotherapeutic agent for the treatment of multidrug-resistant *H. pylori* infections [162]. When WLBU2, a synthetic analogue of the LL-37 peptide (cathelicidin), and ceragenin CSA-13, a synthetic cationic steroid that mimics the activities of a natural cationic antibacterial peptide, were assessed, it was observed that CSA-13, but not LL-37 or WLBU2, retained the antimicrobial activity derived from its ability to compromise bacterial membrane integrity [163]. Recent studies show that although the defensin group of peptides such as HBD-2 and HBD-3 show activity against *Helicobacter* infections, only minimal killing of *H. pylori* was observed, which did not increase by the induction of HBD-2 in *H. pylori*-positive samples [187]. This could be because many antimicrobial peptides undergo proteolytic cleavage by the host digestive components as well as by bacterial enzymes, leading to a major barrier in implementing the use of natural AMPs in treating *Helicobacter* infections. The venom peptide bicarinalin from ants (*Tetramorium bicarinatum*) was found to have a low toxicity in human cells but potent antibacterial activity at the same magnitude as the antibiotics amoxicillin, clarithromycin, metronidazole, and levofloxacin, which are common drugs of choice in therapies against *H. pylori* [164]. Bicarinalin was found to be effective in treating patients with *H. pylori* that was resistant to clarithromycin and levofloxacin but sensitive to metronidazole [164]. It has also been suggested that the use of this bicarinalin as a novel food preservative might also prevent some gastric diseases by acting against *H. pylori* once ingested [188]. The concept of biofilm formation in *H. pylori* is relatively new, and two well-characterised synthetic cationic peptides, IDR-1018 and DJK-5, which show broad spectrum of activity against the biofilms of *K. pneumoniae* differentially affected early- and late-stage biofilm formation in *H. pylori* [147,165]. It was observed that IDR-1018 decreased biofilm formation, although it was not statistically significant, whereas DJK-5 inhibited biofilm formation by the wild-type strain G27 in a dose-dependent manner, and neither peptide has an effect on preformed biofilms or hyperbiofilm-producing strains of *H. pylori* [165]. Human neutrophil peptide 1 is a small cationic peptide (3.44 kDa) belonging to the defensin family, and its recombinant form, produced using the *Pichia pastoris* expression system, shows strong activity against antibiotic-resistant *H. pylori* in vitro and also significantly reduced the colonisation of *H. pylori*, indicating its potential in the treatment of *H. pylori* infections [189].

### 4.8. Campylobacter *spp.*

*Campylobacter* spp. are Gram-negative spiral bacteria that are zoonotic pathogens and the leading cause of bacterial gastrointestinal disease worldwide. Raw/undercooked chicken is considered to be the major source of human *Campylobacter* infection, with other sources that include ruminants, pigs, turkeys, and wild birds [190]. Fluoroquinolone-resistant *Campylobacter* species are identified as high-priority pathogens by the WHO, as fluoroquinolones are the drugs of choice in the treatment of patients with severe *Campylobacter* infections [191]. Biofilms enhance the stress and antimicrobial tolerance of the microorganisms they harbour and play an important role in not only survival but in the farm to fork transmission of *Campylobacter* spp. in humans. Due to the rise in antibiotic resistance, other alternative approaches are urgently needed not only for controlling biofilms but also for improving the efficacy of currently used antibiotics.

The wheat proteins puroindolines of *Triticum aestivum* endosperm occur in two major isoforms, puroindoline A (PinA) and puroindoline B (PinB), and show antibacterial and antifungal properties that are mainly attributed to their characteristic tryptophan-rich domains [166,167]. PinA was shown to inhibit bacterial growth and biofilm formation in *C. jejuni* 81–176 and *C. jejuni* F38011 by damaging the cellular membrane at the concentration of 512 μg/mL and growth at 16–32 μg/mL [167]. The synergistic activity of PinA in combination with erythromycin and ciprofloxacin was observed and was more effective in reducing the growth of *Campylobacter* than the use of antibiotic alone, highlighting its use as a therapeutic agent in health care settings or as preservatives in the agri-food industry [167]. Similarly, the scorpion (*C. suffuses*)-venom-derived AMP Css54, which acts by disrupting the membrane, was shown to exert potent antimicrobial activity on zoonotic bacteria such as *Salmonella typhimurium*, *Listeria monocytogenes*, and *Campylobacter jejuni* [168]. The antimicrobial activity and mode of action of Css54 is similar to melittin (AMP found in honeybees), but it has a lower cytotoxicity than melittin [168]. Some non-ribosomal lipopeptides produced by *Bacillus subtilis* and *Paenibacillus* spp. also exert antimicrobial activity against *Campylobacter* spp., other foodborne bacteria, and phytopathogenic flora [192]. Tridecaptins, a group of cationic linear nonribosomal peptides with a lipid chain on the N-terminus were shown to have potent antimicrobial activity against a variety of Gram-negative bacteria such as *C. jejuni*, *Escherichia coli* O157:H7, and multidrug-resistant *Klebsiella pneumoniae* [169]. Another study demonstrated that clinical and chicken isolates of *C. jejuni* were highly susceptible to chicken cathelicidin-2 (CATH-2) peptide and that *C. jejuni* uses a lipooligosaccharide to protect itself to some extent against host defence peptides by suppressing the expression of CATH-2 levels in chickens, which may be part of the *C. jejuni* immune evasion strategy [170]. Many diverse antimicrobial peptides that have been discovered such as lantibiotics and class IIa-like bacteriocins need to be evaluated further to identify their inhibitory effects against multidrug-resistant *Campylobacter* strains and for their development as preventative and therapeutic agents in controlling foodborne diseases [193].

## 5. Discussion and Conclusions

A strong multi- and cross-disciplinary collaboration of subjects, such as biology, materials science, chemistry, bioinformatics, and molecular informatics, is needed to not just make advances in the development of prospective AMPs but also in solving the problem of AMR, which has emerged as one of the leading public health threats of the 21st century. It is clear that different AMPs work in specific ways with specific modes of action. Hence, the focus should also be on further understanding the correlation between AMPs and various targets, which can aid in improving experimental designs, and on solving the problem of structure and function to obtain more robust data, which can provide evidence-based solutions to global challenges. Advancement in AMPs has led to the development of synthetic or genetically engineered peptides, which can be targeted against a broad range of bacterial hosts. Most of the engineered AMPs are designed to have a broad spectrum of activity, targeting both Gram-positive and -negative pathogens. Although the development of new antimicrobial peptides and peptidomimetics with antimicrobial activity and antibiotics synergy is useful, there is always a problem with bacteria that are primed by antimicrobial peptides and the likelihood of developing tolerance and persistence. For example, Colanic acid is an exopolysaccharide secreted by *E. coli*, *Enterobacter* spp., and by a number of other members of *Enterobacteriaceae*, which is assumed to promote biofilm formation and to protect the organism when exposed to harsh environments, so triggering its expression by sublethal levels of AMPs could potentially catalyse biofilm formation [194]. Antimicrobial agents, including antibiotics and AMPs, are not just wonder drugs but game changers for people who suffer from severe debilitating illnesses and underlying conditions. We also need to focus on the success rate of the clinical application of AMPs, as it will enable us to combat the problem of AMR and other hospital-acquired infections more efficiently. With the breakthrough of a globally accessible, safe, and effective COVID-19 vaccine, modern medicine has become a reality, and it gives everyone hope, but at the same time, it is being gradually undermined as a result of the misuse or overuse of drugs not only in clinical settings but also in the agri-food sector. Although the COVID-19 pandemic has overshadowed the silent pandemic of AMR, which threatens to wreak immense human suffering if not checked, now is the time to confront it. If AMR is not controlled by the prudent use of antibiotics and the development of new antimicrobials and alternative therapies, such as use of phages, novel AMPs, and complementary and alternative medicine, “The future of humanity and microbes will definitely unfold as episodes of Our Wits Versus Their Genes”. We must act now to mitigate the despair and damage that AMR could cause and secure our global health, happiness, and the sustainability of the planet.

## Figures and Tables

**Table 1:** Examples of antimicrobial peptides and their secondary structures, sources, and functions.

References	Antimicrobial Peptide	Secondary Structure	Source	Function/Mode of Action
[17]	Protegrins	α-helix and β-sheet	Porcine	- Antimicrobial activity against *E. coli*, *Listeria monocytogenes*, and *Candida albicans*
[17]	Thanatin	Loop structure	Insect (*Podisus maculiventris*)	- Antibacterial activity (disrupts the bacterial outer membrane)
[19,20]	Tachyplesins	β-sheet	Horseshoe crab	- Antimicrobial activity against multidrug-resistant bacteria via membrane instability mechanism
[22]	Bactenecin	Loop structure	Bovine	- Neurotoxicity
[35]	Human neutrophil peptides (HNP 1-4)	β-sheet	Human	- Microbial killing - Immunomodulative activity
[36,37]	Human β-defensins (HBD 1-4)	β-sheet	Human	- Antimicrobial activity - Affects immune regulation, apoptosis, and wound healing
[38]	Lactoferricin B	Loop structure	Human	- Antimicrobial activity
[39]	Cathelidicin (LL-37)	α-helix	Human	- Antimicrobial activity, - Affects immune regulation, apoptosis, and wound healing
[39]	Human defensin (HD5-6)	α-helix	Human	- Antimicrobial activity - Affects immune regulation, apoptosis, and wound healing
[40]	Magainin-2	α-helix	Frog	- Antimicrobial activity and anticancer properties
[41]	hagfish intestinal AMP (HFIAP)-1, -2, and -3	α-helix	*Myxine glutinosa* (Atlantic hagfish)	- Antimicrobial activity against a number of Gram-positive and -negative bacteria
[42,43]	cathelicidin codCATH	α-helix	Atlantic Cod (*Gadus morhua*)	- Activity against Gram-negative bacteria
[42,43]	rtCATH1 (R146-P181) and rtCATH2 (R143-I178)	α-helix	Rainbow Trout	- Active against *Lactococcus garvieae* and other Gram-negative fish pathogens
[44]	cod-defensin	β-sheet	Atlantic cod	- Shows activity against the Gram-positive microbes *Planococcus citreus* and *Micrococcus luteus*
[45]	Pleurocidin	α-helix	winter flounder (*Pleuronectes americanus*)	- Active against both Gram-positive and -negative pathogens
[46]	Gramicidin	β-sheet	Gram-positive bacteria (*Lactococcus lactis* and *Bacillus brevis*)	- Antimicrobial activity against Gram-positive bacteria
[47,48]	Polymyxin	α-helix	*Paenibacillus polymyxa*	- Active against critically important pathogens (*Enterobacterales*, *A. baumannii*, *P. aeruginosa*, and *S. maltophilia*)
[49]	Bacteriocin	α-helix	Gram-positive bacteria, Gram-negative bacteria	- Antimicrobial activity (act by increasing permeability by forming pores)
[50,51,52]	Colicins	α-helix and β-sheet	*E. coli*	- Channel/pore formation in the cytoplasmic membrane - DNA degradation - Inhibition of murein and lipopolysaccharide biosynthesis
[53]	Peptaibols	α-helix	Fungal	- Membrane disruption
[54]	Plectasin	α-helix	Fungal (*Pseudoplectania nigrella*)	- Inhibitory activity, predominantly against Gram-positive bacteria such as *S. pyogenes*, *C. diphtheriae*, and *S. aureus*
[55]	Micasin	α-helix	*Microsporum canis*	- Broad-spectrum antibacterial activity against *P. aeruginosa* and methicillin-resistant *S. aureus* and affects protein folding
[56]	Melittin	α-helix	European honeybee *Apis mellifera* venom	- Antiviral activity - Anticancer activity
[57,58]	Phage lysins	α-helix	Bacteriophages	- Weakening the peptidoglycan bacterial cell wall, antibiofilm activity
[59]	Holins	α-helix	dsDNA bacteriophages	- Membrane depolarization, endolysin activation, and degradation of peptidoglycans
[60]	HolGH15	α-helix	*S. aureus* bacteriophage GH15	- Antibacterial activity against *L. monocytogenes*
[61]	Cecropin A	α-helix	Cecropia moth (*Hyalophora cecropia*) and bees	- Activity against different inflammatory diseases and cancers - Antimicrobial effect, mainly against Gram-negative bacteria
[62]	Jellein	α-helix	Honey bee royal jelly	- Antibacterial and antifungal activity

**Table 2:** Antimicrobial peptides effective against bacterial biofilm-producing Gram-positive and Gram-negative pathogens.

Bacterial Species	Antimicrobial Peptides	References
*Enterococcus faecalis*	C16-KGGK KP and L18R (antifungal peptides) Buwchitin P-113 Bip-P-113 SAAP-148 (LL-37 derivative)	[136] [137] [138] [139] [140]
*Staphylococcus aureus*	NA-CATH:ATRA1-ATRA1 human lysozyme-derived peptide (LP9) polymyxin B gallidermin phage-derived bacteriocins SAAP-148 (LL-37 derivative)	[141] [142] [142] [142] [143,144] [140]
*Klebsiella pneumoniae*	Analog PepC (A6, A12, and A19) cathelicidin-derived peptide D-11 DJK-6 IDR-1018 citropin 1.1 CAMEL SAAP-148 (LL-37 derivative)	[145] [146] [147] [148] [149] [140]
*Acinetobacter baumannii*	Cec4 LL-37 SMAP-29 Tilapia Piscidin 4 (TP4) Combination of melittin and doripenem plyAB1 ply6A3 plyF307 SAAP-148 (LL-37 derivative)	[150] [151] [152] [152] [153] [154] [155] [140]
*Pseudomonas aeruginosa*	Lactoferrin LL-37 LysPA26 SAAP-148 (LL-37 derivative)	[129] [156] [157]
*Enterobacter* spp.	BMAP-27B SMAP-29D SAAP-148 (LL-37 derivative)	[158] [158] [140]
*Helicobacter pylori*	Cathelicidin mCRAMP Tilapia Piscidin 4 (TP4) ceragenin CSA-13 bicarinalin IDR-1018 DJK-5	[159,160,161] [162] [163] [164] [165] [165]
*Campylobacter* spp.	puroindoline A (PinA) Css54 (Scorpion-venom-derived AMP) Tridecaptins chicken cathelicidin-2 (CATH-2)	[166,167] [168] [169] [170]
